# Adrenomedullin induces cisplatin chemoresistance in ovarian cancer through reprogramming of glucose metabolism

**DOI:** 10.2478/jtim-2023-0091

**Published:** 2023-07-05

**Authors:** Lei Dou, Enting Lu, Dongli Tian, Fangmei Li, Lei Deng, Yi Zhang

**Affiliations:** Department of Gynecology, the First Affiliated Hospital of China Medical University, Shenyang 110001, Liaoning Province, China

**Keywords:** adrenomedullin, pyruvate kinase isozyme type M2, glucose metabolism reprogramming, cisplatin chemoresistance

## Abstract

**Background and Objectives:**

The metabolic network of cancer cells has been reprogrammed – relying more on aerobic glycolysis to gain energy, which is an important reason for drug resistance. Expression of adrenomedullin (ADM) in ovarian cancer tissues is related to resistance to platinum-based drugs. In view of this, we intended to investigate the correlation between ADM and glucose metabolism reprogramming of tumor cells to clarify the possible mechanism of ADM-induced ovarian cancer cisplatin resistance through glucose metabolism reprogramming.

**Methods:**

Epithelial ovarian cancer (EOC) cell viability and apoptosis were determined. Different gene expression and protein levels were detected by real-time revere transcription polymerase chain reaction and western blotting. Oxygen consumption rate (OCR) and extracellular acidification rates (ECARs) were measured.

**Results:**

*ADM* expression was upregulated in cisplatin-resistant EOC cells. ADM attenuated cisplatin-inhibited cell survival and cisplatin-induced apoptosis in sensitive EOC cells; knockdown of ADM enhanced cisplatin chemosensitivity of cisplatin-resistant EOC cells. ADM enhanced glycolysis in cisplatin-sensitive EOC cells; knockdown of ADM significantly inhibited glycolysis in cisplatin-resistant EOC cells. ADM significantly upregulated pyruvate kinase isozyme type M2 (PKM2) protein level, the key enzyme during glycolysis; PKM2 inhibitor significantly abolished the ADM-improved cell survival and ADM-inhibited apoptosis.

**Conclusion:**

ADM promoted proliferation and inhibited apoptosis of ovarian cancer cells through reprogramming of glucose metabolism, so as to promote cisplatin resistance. The study is expected to identify multidrug resistance markers of ovarian cancer and provide a target for the prevention and treatment of ovarian cancer, which is important for clinical translational research.

## Introduction

Ovarian cancer is one of the most common malignancies of the female reproductive system. It ranks third in morbidity after cervical cancer and uterine body cancer, but ranks first in mortality among gynecological malignancies.^[1]^ Because the ovaries are located in the deep pelvic cavity, early symptoms of ovarian cancer are hidden and there is a lack of accurate early diagnostic methods. So, most patients are diagnosed late. Clinically, tumor cell reduction combined with chemotherapy based on cisplatin and paclitaxel is the main treatment, but the recurrence rate of this regimen is as high as 70% two years after surgery.^[2]^ Studies have shown that the resistance to platinum-based drugs gradually acquired by patients is the main reason for the high recurrence of ovarian cancer in the course of platinum-based drug treatment,^[3, 4]^ and this resistance also leads to decreased sensitivity to radiotherapy or other drugs with different pharmacological mechanisms, which seriously affects the clinical efficacy of treatment of ovarian cancer. Therefore, it is important to study the mechanism of platinum-based drug resistance in ovarian cancer.

There are many mechanisms for the formation of multidrug resistance in tumors, which are currently believed to be mainly related to increased drug excretion by the tumor cells, decreased drug intake, enhanced activity of drug-metabolizing enzymes, dysregulation of DNA damage and repair, blocked apoptosis, autophagy, and changes in cell microenvironment.^[5]^ Recent studies have shown that reprogramming of glucose metabolism in tumor cells also plays an important role in the multidrug resistance of ovarian cancer.^[6]^ In normal tissue cells, 90% of adenosine triphosphate (ATP) comes from mitochondrial oxidative phosphorylation and only 10% comes from glycolysis.^[7]^ In contrast, tumor cells do not utilize mitochondrial oxidative phosphorylation, even in aerobic conditions, but instead utilize aerobic glycolysis, known as the Warburg effect, which is an important manifestation of glycometabolic reprogramming in tumor cells.^[8]^

Adrenomedullin (ADM) is a peptide that was originally isolated from pheochromocytoma and is widely distributed in the body. It is expressed in normal adrenal medulla, heart, lungs, kidneys, vascular endothelial cells, vascular smooth muscle cells, and other tissues.^[9]^ More and more studies have found that ADM is expressed in many tumor cell lines and malignant tissues and is closely related to the occurrence, development, and drug resistance of tumors.^[10, 11, 12, 13, 14, 15, 16, 17, 18]^ ADM is an autocrine growth factor of tumor cells, and its expression can be detected in tumor cells from various tissues (adrenal gland, bone marrow, breast, cartilage, colon, lungs, nervous system, prostate, and ovaries).^[19]^ Increase in ADM level in the serum of patients with many types of tumors can also be detected, and the increase is related to the malignant degree of tumors. After surgical resection of the primary tumor, the serum ADM level can be restored to normal.^[12, 19]^ Studies have shown that ADM can promote the proliferation of tumor cells, reduce the apoptosis rate of tumor cells, help tumor cells escape immune surveillance, and promote tumor angiogenesis.^[19, 20]^ There are significant differences in the expression of ADM in malignant and benign ovarian tumor tissues and normal ovarian tissue, suggesting that ADM is mainly produced by the ovarian malignant tumor tissues. ADM can be used as an indicator of malignant degree and poor prognosis of ovarian cancer, which is helpful for the diagnosis of high-risk patients in the early stage.^[21]^ However, the expression of ADM in epithelial ovarian cancer (EOC) has only recently been studied and further studies are needed.

In the present study, we analyzed the correlation between ADM and glucose metabolism reprogramming of cisplatin-resistant ovarian cancer and the underlying mechanism.

## Materials and Methods

### Materials

Cisplatin-sensitive A2780 EOC cells and cisplatin-resistant A2780cis cells were purchased from the European Collection of Authenticated Cell Cultures (ECACC; Porton Down, Salisbury, UK). Roswell Park Memorial Institute (RPMI) 1640 medium was purchased from Sigma Aldrich (St. Louis, MO, USA). Fetal bovine serum (FBS) was purchased from HyClone (Logan, UT, USA). Recombinant human ADM (full length 1–53 amino acids) and its receptor antagonist ADM22-52 were purchased from Phoenix Pharmaceuticals (Belmont, CA, USA). Mouse-anti-β-actin antibody was purchased from Santa Cruz Biotechnology (Santa Cruz, CA, USA). All other antibodies were purchased from Cell Signaling Technology (Beverly, MA, USA). IRDye-conjugated affinity-purified anti-rabbit and anti-mouse IgGs were purchased from Rockland (Gilbertsville, PA, USA). Trizol reagent and the reverse transcription (RT) system were purchased from Invitrogen Inc. (Grand Island, NY, USA). All other chemicals and drugs were purchased from Sigma Chemical (St. Louis, MO, USA).

### Cell culture

Cisplatin-sensitive A2780 cells and cisplatin-resistant A2780cis cells were cultured in RPMI 1640 containing 10% FBS and penicillin/ streptomycin (100 U/mL) in a humidified 37℃ incubator. When confluent, cells were treated with ADM. For the inhibition experiments, cells were pretreated with ADM22-52 for 1 h before stimulation with ADM.

### Construction of expression vector and cell transfection

The short hairpin (sh)RNA sequences targeting *ADM* or scramble sequences were designed as previously reported.^[22]^ The shRNA sequences were submitted to BLAST (http://www.ncbi.nlm.nih.gov/blast/) to ensure their specificity of targeting. The pRNA-U6.1/Neo was selected as the RNAi expression vector. The double-stranded shDNA was annealed, ligated into the restriction sites between *Bam*HI and *Xho*I in pRNA-U6.1/Neo with T4 DNA ligase, and then transfected into *Escherichia coli* DH5α. Resistance screening, agarose gel electrophoresis, and DNA sequencing were used to check the recombinant plasmid, named pRNA-shADM.

For transfection, cells (5 × 104) were plated in six-well plates and allowed to adhere for 24 h. Plasmid DNA was transfected into cells using Lipofectamine^™^ 2000 (Thermo Fisher Scientific, Waltham, MA, USA). Cells were transfected using 2 μg DNA mixed with 5 μL Lipofectamine in 1 mL medium without serum or antibiotics. The cells were incubated for 24 h, and 1 mL medium containing 20% serum was added to each well. The cells were cultured and screened in medium containing 10% serum and 400 μg/mL G418 for at least 3 weeks. Stable transfectants were formed.

### Cell proliferation detected with Cell Counting Kit-8

The Cell Counting Kit‑8 (CCK‑8) assays were used to determine the effect of ADM on SKOV3 cell viability. Cells (103/well) were incubated in 96‑well plates overnight, starved in serum‑free medium for 24 hm and treated with indicated reagents. Cells were incubated with 10 μL CCK-8 solution (Dojindo Molecular Technologies, Kumamoto, Japan) for 1 h and the absorbance was measured at 450 nm.

### RNA extraction and reverse transcription-polymerase chain reaction analysis

Total RNA was isolated using TRIzol reagent. Total RNA (2 μg) was reverse transcribed using an RT system. One microliter of the reaction mixture was subjected to reverse transcription-polymerase chain reaction (RT-PCR). The polymerase chain reaction (PCR) primers are listed in Table 1. All amplification reactions were performed under the following conditions: 95°C for 5 min, followed by 35 cycles at 95°C for 30 s, 58°C for 30 s, and 72°C for 60 s. For the quantitative real-time RT-PCR analysis, the amount of PCR products formed in each cycle was evaluated on the basis of SYBR Green I (Solarbio, Beijing, China) fluorescence. Results were analyzed with Stratagene Mx3000 software, and mRNA levels were normalized with respect to the levels of glyceraldehyde-3-phosphate dehydrogenase (GAPDH) in each sample. The primers used in this study were as follows: human *ADM*, 5′-TCCCCCTATTTTAAGACGTGAATG-3′ and 5′-CATGCACACAAACACACTCACAT-3′and human *GAPDH*, 5′- GCACCGTCAAGGCTGAGAAC-3′ and 5′-TGGTGAAGACGCCAGTGGA-3′.

### Preparation of cytosolic proteins and western blotting

Following treatment, the cells were packed by centrifugation at 200 ×*g* for 3 min and homogenized in ice-cold fractionation buffer. The cell lysate was incubated on ice for 15 min and then centrifuged at 20,000 ×*g* for 30 min at 4°C. The cytosolic fraction was collected and subjected to 10% SDS-PAGE. Protein concentrations were determined by BCA Protein Assay Kit (Pierce, Rockford, IL, USA). The proteins were transferred to a polyvinylidene fluoride membrane. The membrane was incubated successively with 5% bovine serum albumin in Tris Tween buffered saline at room temperature for 1 h, followed by incubation overnight at 4°C with the individual primary antibody. Specific reaction was detected using IRDye-conjugated second antibody and visualized using the Odyssey infrared imaging system (LI-COR Biosciences, Lincoln, NE, USA). Quantification of image density in pixels was performed using the Odyssey infrared imaging system (LI-COR Biosciences).

### Apoptosis analysis

Cells were cultured in 96-well plates with or without ghrelin (10^-9^ mol/L) stimulation. Apoptosis was assessed by measuring cysteine aspartic acid-specific protease (caspase) 3/7 activity with the use of the Caspase-Glo^®^ 3/7 Assay Kit (Promega, Madison, WI, USA).

### Extracellular flux analysis

Oxygen consumption rate (OCR) and extracellular acidification rates (ECARs) were measured on a Seahorse XF24 extracellular flux analyzer (Seahorse Bioscience, Billerica, MA, USA). Cells were seeded into a Seahorse 24-well plate coated with poly-l-lysine hydrobromide. Before assay, the cells were incubated with Seahorse XF base medium supplemented with 10 mmol/L glucose, 1 mmol/L pyruvate, and 2 mmol/L glutamine in a CO^2^-free incubator equilibrated for 1 h. During assay, 1 mmol/L oligomycin, 1 mmol/ L carbonyl cyanide *p*-trifluoromethoxyphenylhydrazone, 1 mmol/L rotenone, and 1 mmol/L antimycin A were added sequentially. The data were analyzed with Wave software and the XF Mito/Glycolysis Stress Test Report Generator (Seahorse Bioscience).

### Detection of lactate in cell culture medium

The cells were inoculated into the 24-well plates at 1 × 105/ well. Each group had five duplicated wells. The cell culture medium was collected, and then, the level of lactate was measured according to the operation instructions of the enzyme-linked immunosorbent assay (ELISA) kit (Keshun Bio, China).

### Statistical analysis

Quantitative data are presented as the means ± standard error of mean (SEM) determined from the indicated number of experiments. Statistical analysis was based on Student’s *t*-test for comparison of two groups or one-way analysis of variance for multiple comparisons. *P* < 0.05 was used to determine statistical significance.

## Results

### ADM expression is upregulated in cisplatin-resistant EOC cells

**Figure 1:**
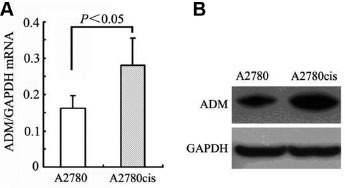
Expression of ADM in ovarian epithelial carcinoma cells. (A) ADM mRNA expression and (B) ADM protein levels in cisplatin-sensitive control A2780 cells and cisplatin-resistant A2780cis cells. GAPDH was used as an internal control. Data are means ± SEM. ADM: adrenomedullin; GAPDH: glyceraldehyde-3-phosphate dehydrogenase; SEM: standard error of the mean.

We examined the expression of *ADM* in epithelial ovarian carcinoma cells. Compared to cisplatin-sensitive control A2780 cells, the levels of *ADM* mRNA in cisplatin-resistant A2780cis cells increased significantly (Figure 1A). A similar increment was observed for ADM at the protein level (Figure 1B). These results confirmed that *ADM* expression was upregulated in cisplatin-resistant cells.

### ADM inhibited cisplatin chemosensitivity of EOC cells

We confirmed the cisplatin chemosensitivity of EOC cells.

**Figure 2:**
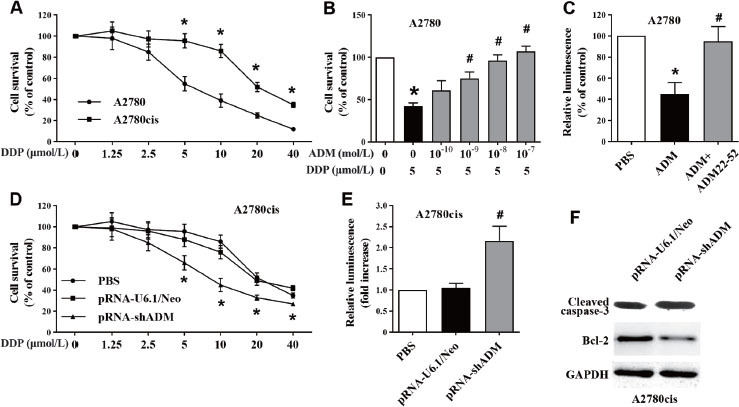
Effect of ADM on cisplatin chemosensitivity of EOC cells. (A) Cisplatin-sensitive control A2780 cells and cisplatin-resistant A2780cis cells were treated with cisplatin (DDP) supplied for 72 h, and viability was detected using CCK‑8 assays. (B) Sensitive A2780 cells were treated with cisplatin (5 μmol/L) and ADM for 72 h, and viability was detected using CCK‑8 assays. (C) Caspase-3/7 activity detected in A2780 cells treated with ADM. Cells were pretreated with ADM22-52 for 1 h and then with ADM (10^-8^ mol/L) for 72 h. Data are means ± SEM from four separate experiments. ^*^P < 0.05 *versus* control; ^#^*P* < 0.01 *versus* cisplatin treatment alone. (D–F) ADM was knocked down in A2780cis cells through transfection with ADM-targeted recombinant plasmid (pRNA-shADM) or control (pRNA-U6.1/Neo), and viability (D), caspase-3/7 activity (E), cleaved caspase-3 and Bcl-2 protein levels (F) were detected. Data are means ± SEM from four separate experiments. ^*^P < 0.05 *versus* PBS treatment; ^#^*P* < 0.01 *versus* pRNA-U6.1/Neo transfection. ADM: adrenomedullin; EOC: epithelial ovarian cancer; PBS: phosphate buffer solution; CCK-8: Cell Counting Kit‑8; SEM: standard error of the mean.

The survival rate of A2780 cells significantly decreased under cisplatin treatment at 5–40 μmol/L, whereas the cell survival rate of A2780cis cells significantly decreased under cisplatin treatment at 20–40 μmol/L (Figure 2A). We chose 5 and 20 μmol/L cisplatin for treatment of A2780 and A2780cis cells. Inhibition of cell survival by cisplatin treatment was significantly attenuated by ADM (Figure 2B) in cisplatin-sensitive A2780 cells, as well as apoptosis (Figure 2C), which was attenuated by ADM22-52, the receptor competitor of ADM, indicating inhibition of cisplatin chemosensitivity. In cisplatin-resistant A2780cis cells, knockdown of ADM significantly abolished the resistance to cisplatin (Figure 2D), as well as increasing apoptosis (Figure 2E and F). These results indicated that ADM inhibited cisplatin chemosensitivity of EOC cells through its receptor.

### ADM induced reprogramming of glucose metabolism in EOC cells

**Figure 3:**
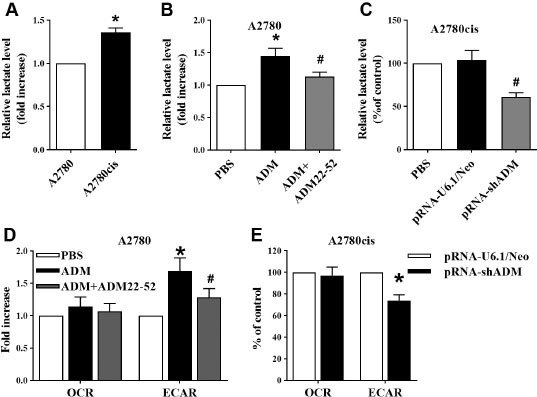
Role of ADM in aerobic glycolysis in EOC cells. (A) Increased lactate levels (indicators of glycolysis) in cisplatin-resistant A2780cis cells. (B) Effects of ADM on lactate levels in A2780 cells. (C) Knockdown of ADM significantly abolished glycolysis in A2780cis cells. (D) ADM increased ECAR levels (indicator of glycolysis), but not OCR levels (indicator of oxidative phosphorylation) in A2780 cells. (E) Knockdown of ADM significantly decreased ECAR levels in A2780cis cells. All data are shown as mean ± SEM (*n* = 4). ^*^P < 0.05 *versus* control; ^#^*P* < 0.01 *versus* ADM treatment. ADM: adrenomedullin; EOC: epithelial ovarian cancer; PBS: phosphate buffer solution; OCR: oxygen consumption rate; ECAR: extracellular acidification rate; SEM: standard error of the mean.

Emerging evidence has found that tumor cells can rapidly switch glucose metabolism to aerobic glycolysis, a phenomenon known as the Warburg effect, to rapidly provide ATP and metabolic intermediates for supporting proliferation. Lactate secretion is one part of the classical glycolytic index. We found significantly higher level of lactate in the medium of A2780cis cells (Figure 3A), indicating increased glycolysis in cisplatin-resistant cells. ADM induced secretion of lactate in EOC medium (Figure 3B). Knockdown of ADM significantly abolished glycolysis in A2780cis cells (Figure 3C). Similar results were found for change of cellular ECAR (indicative of glycolysis), but not OCR (indicative of oxidative phosphorylation) (Figure 3D and E). These results indicated that ADM induced reprogramming of glucose metabolism in EOC cells.

### Reprogramming of glucose metabolism mediated ADM-induced cisplatin chemoresistance in EOC cells

**Figure 4:**
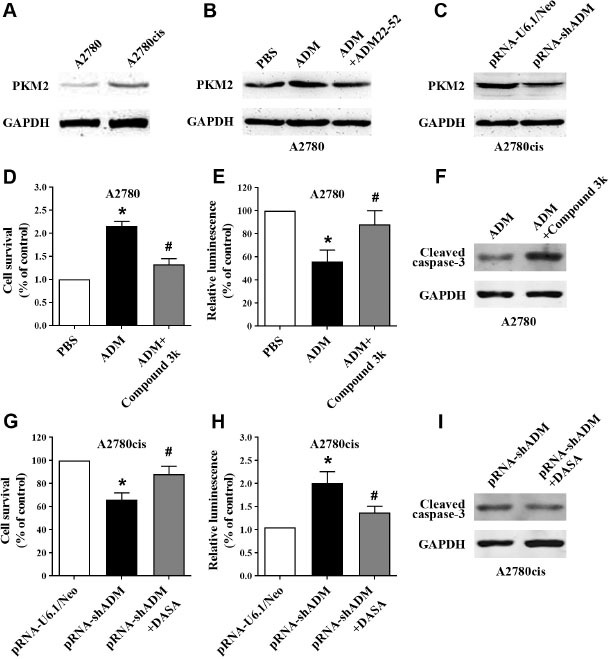
Reprogramming of glucose metabolism mediated ADM-induced cisplatin chemoresistance in EOC cells. (A–C) PKM2 protein levels were detected by western blotting in cisplatin-sensitive control A2780 cells and cisplatin-resistant A2780cis cells. Role of PKM2 inhibitor compound 3k in ADM-stimulated cell survival (D) and apoptosis (E and F). Role of PKM2 activator DASA in ADM knockdown-inhibited cell survival (G) and ADM knockdown-stimulated apoptosis (H and I). ^*^P < 0.05 *versus* control; ^#^*P* < 0.01 *versus* ADM treatment. ADM: adrenomedullin; EOC: epithelial ovarian cancer; PBS: phosphate buffer solution; PKM2: pyruvate kinase isozyme type M2; GAPDH: glyceraldehyde-3-phosphate dehydrogenase.

Previous studies have found that the activation of pyruvate kinase isoenzyme M2 (PKM2) is involved in the aerobic glycolysis of tumor cells. The protein level of PKM2 is significantly higher in cisplatin-resistant A2780cis cells, indicating the key role of glucose metabolism reprogramming in EOC chemosensitivity (Figure 4A). Exogenous administration of ADM significantly increased PKM2 protein level (Figure 4B), which was abolished by ADM22-52; knockdown of endogenous ADM significantly reduced PKM2 protein level (Figure 4C), indicating that PKM2 is the downstream molecule of ADM signaling. In cisplatin-sensitive A2780 cells, compound 3k, a PKM2 inhibitor, significantly abolished the ADM-improved cell survival (Figure 4D) and ADM-inhibited apoptosis (Figure 4E and F), whereas DASA, the PKM2 activator, significantly restored cell survival (Figure 4G) and inhibited apoptosis (Figure 4H and I) induced by lack of endogenous ADM. These results showed that reprogramming of glucose metabolism mediated ADM-induced cisplatin chemoresistance in EOC cells.

## Discussion

**Figure 5:**
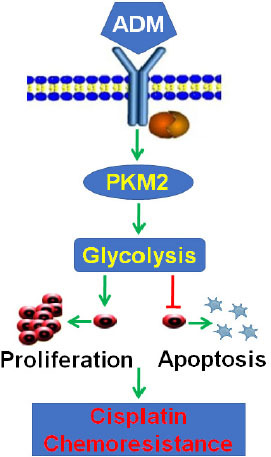
Illustration of putative signaling pathways. ADM promoted proliferation and inhibited apoptosis of ovarian cancer cells through reprogramming of glucose metabolism, so as to promote cisplatin resistance. ADM: adrenomedullin; PKM2: pyruvate kinase isozyme type M2.

The present study demonstrated that ADM can induce cisplatin chemoresistance in human ovarian epithelial carcinoma cells through reprogramming of glucose metabolism via upregulation of PKM2 and subsequently contribute to cancer prevention and therapy. This conclusion is supported by the following observations: (1) *ADM* expression was upregulated in cisplatin-resistant EOC cells; (2) ADM attenuated cisplatin-inhibited cell survival and cisplatin-induced apoptosis in sensitive EOC cells; (3) knockdown of ADM enhanced cisplatin chemosensitivity of cisplatin-resistant EOC cells; (4) ADM enhanced glycolysis in cisplatin-sensitive EOC cells; (5) knockdown of ADM significantly inhibited glycolysis in cisplatin-resistant EOC cells; and (6) ADM significantly upregulated PKM2 protein level, the key enzyme during glycolysis. Reprogramming of glucose metabolism mediated ADM-induced cisplatin chemoresistance in EOC cells. To the best of our knowledge, this is the first study to demonstrate the involvement of glucose metabolism reprogramming in ADM-mediated modulation of cisplatin chemoresistance in ovarian epithelial carcinoma (Figure 5). Anti-ADM therapy with ADM-neutralizing antibodies, ADM receptor antagonists, or ADM receptor interference might be a promising strategy to be explored in ovarian cancer, not only as an antiangiogenic alternative in the context of acquired resistance to vascular endothelial growth factor (VEGF) treatment, but also as a potential metabolism-related approach.

Due to the unlimited proliferative ability of tumor cells, most solid tumors and their metastases are in a hypoxic state. The significance of glycolytic reprogramming of tumor cells lies in the following.^[23–29]^ Firstly, it increased cell tolerance to hypoxia, making the cancer cells more resistant to nest-loss apoptosis. Secondly, the rapid proliferation of tumor cells requires not only ATP, but also biomolecules used to form various cellular structures. Studies have shown that the metabolic abnormality of tumor cells is not a simple adjustment of a single metabolic pathway, but a subversive change in the entire cellular metabolic network. Metabolic reprogramming aims to drive limited nutrients or intermediate metabolites into various biosynthetic pathways to support the vigorous anabolism of tumor cells. For example, the intermediate products of glycolytic pathway, 6-phosphoric acid-glucose, 6-phosphoric acid-fructose, and 3-phosphoric acid-glyceraldehyde, can be used for *de novo* synthesis of nucleic acids. Phosphoric acid metabolites upstream of pyruvate accumulate in tumor cells and can be used in the synthesis of fatty acids and nucleic acids to enable tumor cell survival. Thirdly, lactic acid, the end product of glycolysis, can decompose and destroy the cell matrix around tumor cells, change the tumor microenvironment, and promote migration of tumor cells.

In the glycolytic metabolic pathway, some key enzymes are involved in drug resistance of tumor cells, such as hexokinase (HK)^[30]^ and phosphofructokinase (PFK),^[31]^ pyruvate kinase (PK),^[32, 33, 34]^ lactate dehydrogenase (LDH),^[35, 36, 37]^ and glycerol phosphate dehydrogenase (GPDH). The common feature of these key enzymes is that they are all transformed into embryonic subtypes to support the large-scale metabolism and synthesis required for cell proliferation and provide various benefits for cancer cells, such as preventing apoptosis, increasing invasiveness, and increasing drug resistance. Experimental studies have shown that the composition ratio of PK subtypes in a variety of drug-resistant cell lines cultured *in vitro* has changed.^[38]^ Expression of HK-II in 5-fluoruracil-resistant gastric cancer cells (AGS) is upregulated.^[39]^ The expression of 6-phosphofructo-2-kinase/fructose-2,6-biphosphatase 3 (PFKFB3) is upregulated in paclitaxel-resistant breast cancer cells (MCF-7).^[40]^

Pyruvate dehydrogenase complex (PDHC) is a key enzyme that links aerobic oxidation of sugar with the tricarboxylic acid cycle and oxidative phosphorylation.^[41]^ Decrease or deletion of this enzyme leads to cellular generation. The pathway is transformed from oxidative phosphorylation to glycolysis.^[42]^ Analysis of 248 ovarian cancer specimens showed low expression of pyruvate dehydrogenase A1 in ovarian cancer cells and poor prognosis of patients.^[43]^ Another study on the energy metabolism of ovarian cancer cells indicated that drug-resistant ovarian cancer cells have a more active energy metabolism phenotype, and this promotes cell survival, invasion, and drug resistance, rather than promoting tumor cell proliferation.^[44]^ In addition, both paclitaxel-resistant ovarian cancer cells (ES-2) and cisplatin-resistant ovarian cancer cells (C13) show enhanced aerobic fermentation. Inhibition of glycolysis-related enzymes or silencing related genes can ameliorate the drug resistance of ovarian cancer cells. Treatment of ovarian cancer cell lines with mixed inhibitors of various enzymes in the glycolytic pathway can effectively reduce the production of lactic acid and increase the drug sensitivity of cells.^[45]^ Therefore, exploring the reprogramming pathway of glucose metabolism of tumor cells and regulating energy metabolism can improve the drug sensitivity of tumor cells, including ovarian cancer cells. In-depth study using animal models is needed to verify the essential role of the reprogramming pathway of glucose metabolism *in vivo*. Furthermore, confirmation in clinical specimens is also necessary.

In conclusion, ADM promoted proliferation and inhibited apoptosis of ovary cancer cells through reprogramming of glucose metabolism, so as to promote cisplatin resistance. The present study is expected to identify multidrug resistance markers of ovarian cancer and provide an intervention target for the prevention and treatment of ovarian cancer, which has the potential for clinical translational research.
